# Racial disparities in opioid prescription and pain management among breast cancer survivors

**DOI:** 10.1002/cam4.5755

**Published:** 2023-03-14

**Authors:** Sunny Jung Kim, Reuben P. Retnam, Arnethea L. Sutton, Megan C. Edmonds, Dipankar Bandyopadhyay, Vanessa B. Sheppard

**Affiliations:** ^1^ Department of Health Behavior and Policy, School of Medicine Virginia Commonwealth University Richmond Virginia USA; ^2^ Massey Cancer Center Virginia Commonwealth University Richmond Virginia USA; ^3^ Takeda Pharmaceuticals Virginia Massachusetts USA; ^4^ Division of General Internal Medicine Icahn School of Medicine at Mount Sinai New York New York USA; ^5^ Department of Biostatistics, School of Medicine Virginia Commonwealth University Richmond Virginia USA

**Keywords:** cancer patients, opioids, pain management disparity

## Abstract

**Background:**

We examined whether there are racial disparities in pain management, opioid medicine prescriptions, symptom severity, and quality of life constructs in breast cancer survivors.

**Methods:**

We conducted a secondary analysis of longitudinal data from the Women's Hormonal Therapy Initiation and Persistence (WHIP) study (*n* = 595), a longitudinal study of hormonal receptor‐positive breast cancer survivors. Upon study enrollment, patients completed a survey assessing an array of psychological, behavioral, and treatment outcomes, including adjuvant endocrine therapy (AET)‐induced symptoms, and provided a saliva biospecimen. Opioid prescription records were extracted from the health maintenance organizations (HMOs) pharmacy database. The final analytic sample included women with complete HMO pharmacy records for 1 year.

**Results:**

There were 251 eligible patients, of which 169 (67.3%) were White. The average age was 61.09 years old (*SD* = 11.07). One hundred seventy‐two patients (68.5%) had received at least one opioid medication and 37.1% were prescribed opioids longer than 90 days (*n* = 93). Sixty‐four Black patients (78%) had a record of being prescribed with opioids compared to 64% of White patients (*n* = 108, *p* = 0.03). Black patients reported worse vasomotor, neuropsychological, and gastrointestinal symptoms, as well as lower quality of life and greater healthcare discrimination than White patients (*p's* < 0.05). Black patients were more likely to be prescribed opioids for 90 days or longer compared to White patients, when controlling for age, marital status, income, body mass index (BMI), cancer stage, and chemotherapy status (adjusted Odds Ratio = 2.72, *p* = 0.014).

**Conclusion:**

Findings indicate that there are racial differences in opioid prescriptions supplied for pain management and symptomatic outcomes. Future research is needed to understand the causes of disparities in cancer pain management and symptomatic outcomes.

## Graphical abstract

We found racial disparities in opioid prescriptions and symptomatic outcomes among cancer patients. Black cancer patients were more likely to be prescribed opioids compared to White patients. Black patients reported worse vasomotor, neuropsychological, and gastrointestinal symptoms, as well as lower quality of life than White patients.

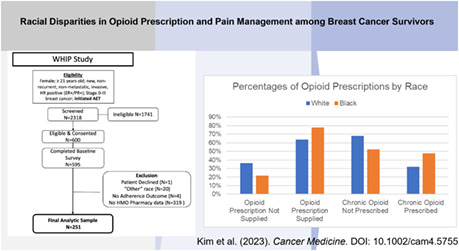

## 1 INTRODUCTION

Cancer survivors experience post‐treatment complications and morbidities. Pain is one of the prevalent health problems in cancer survivors resulting from the tumor, exposure to treatment, or other medical comorbidities. Chronic pain is as prevalent as 40% among cancer survivor populations,^1^ with the most common form of chronic pain being neuropathic pain.^2^ Chronic pain in cancer survivors can last for months to years, which can significantly reduce quality of life.^3^, ^4^ While nonpharmacologic therapies alone may be used to treat mild cancer‐related pain, pharmacologic treatment that involves opioids is often used as part of therapy for cancer‐related pain.^5^, ^6^

According to the National Cancer Institute, a cancer survivor is an individual who remains alive during and after overcoming cancer; thus, cancer survivorship starts from the time of diagnosis and lasts until the end of life.^7^ Cancer survivors have a high rate of opioid use.^5^ Younger age, unemployment at the time of cancer diagnosis, increased comorbidity, and prior alcohol use were associated with increased adjusted odds of opioid use.^8^ There are growing concerns about the risks of long‐term opioid use and racial disparities in pain management among cancer survivors.^4^ It is reported that cancer patients of racial and ethnic minorities showed greater comorbidities and encounter more barriers to receiving quality of care than other patient groups.^9^ Black and Hispanic cancer survivors are more likely to experience barriers to receiving pain treatment and limited access to healthcare due to inability to afford the cost of medications even with insurance coverage.^10^ A recent systematic review indicates that cancer patients of racial and ethnic minorities receive less optimal care for acute and chronic pain than do Whites even after controlling for age, sex, and pain severity.^6^, ^11^ Cancer survivors with racial and ethnic minorities consistently underreported pain intensity, which, in part, contributed to unmet needs of pain management.^2^, ^12^, ^13^ Cancer survivors of racial and ethnic minorities were also more likely than Whites to report low quality of life caused by untreated pain.^14^ Understanding the associations between opioid treatment exposure and health conditions between racial groups is critical from a health equity standpoint to improve the quality of care of individuals of racial minorities.^6^, ^11^

The severity of chronic pain is relatively higher in certain cancer subpopulations, such as breast cancer survivors.^3^, ^15^ Breast cancer survivors prescribed with adjuvant endocrine therapy (AET) are at an elevated risk for pain, comorbidities, and adverse effects (e.g., vasomotor symptoms).^16^ Quality of life and symptomatic outcomes are shown to vary by pain severity and pain management.^14^ Although opioid therapy is widely used to treat moderate to severe cancer‐related pain, given the reported patterns of racial disparities in cancer‐related health outcomes, it is critical to examine whether AET breast cancer survivors of racial and ethnic minorities are more likely to experience adverse effects of opioid use than their White counterparts, and how those adverse effects are related to the duration of opioids prescribed.^17^ Despite the accumulating evidence of racial disparities in pain management and differing treatment outcomes by chronic opioid use, there is a dearth of research directly investigating how various symptomatic outcomes and quality of life among AET breast cancer survivors differ by race and exposure to opioids prescribed. To understand treatment outcomes and characteristics of cancer survivors who are prescribed opioid medicines for a long period of time, we examined a wide range of symptomatic outcomes, quality of life, and psychological parameters across racial groups as well as opioid prescription status (e.g., nonopioid prescribed, chronic opioids prescribed). We hypothesized that Black AET breast cancer survivors are more likely to report worse symptomatic outcomes, low quality of life, and greater pain than Whites, and that these health conditions are worse among those who were prescribed opioids for a long term versus those with non‐ or short‐term exposure to opioid medicines.

## 2 METHODS

### 2.1 Data collection

The study is a secondary data analysis from the Women's Hormonal Therapy Initiation and Persistence (WHIP) study, a longitudinal study that sought to understand endocrine therapy adherence and patient outcomes in breast cancer survivors.^18^, ^19^ In this study, women with hormone receptor‐positive (HR+) breast cancer were recruited from integrated health systems, communities, and organizations in Washington D.C.; Atlanta, GA; and Detroit, MI. The study eligibility criteria included self‐identified as either African American or White, ≥21 years of age, diagnosed with HR+ (estrogen‐receptor‐positive [ER+] and/or progesterone‐receptor‐positive [PR+]) breast cancer within 1 year of study enrollment, between stage 0 and III breast cancer, and prescribed adjuvant endocrine therapy (AET), and having filled a prescription script based on pharmacy records for any type of AET (e.g., tamoxifen) within 1‐year postdiagnosis. Medical and pharmacy records were provided by each institution to capture the type of AET that patients were prescribed and confirm that women had current AET prescription at the time of survey.

**FIGURE 1:**
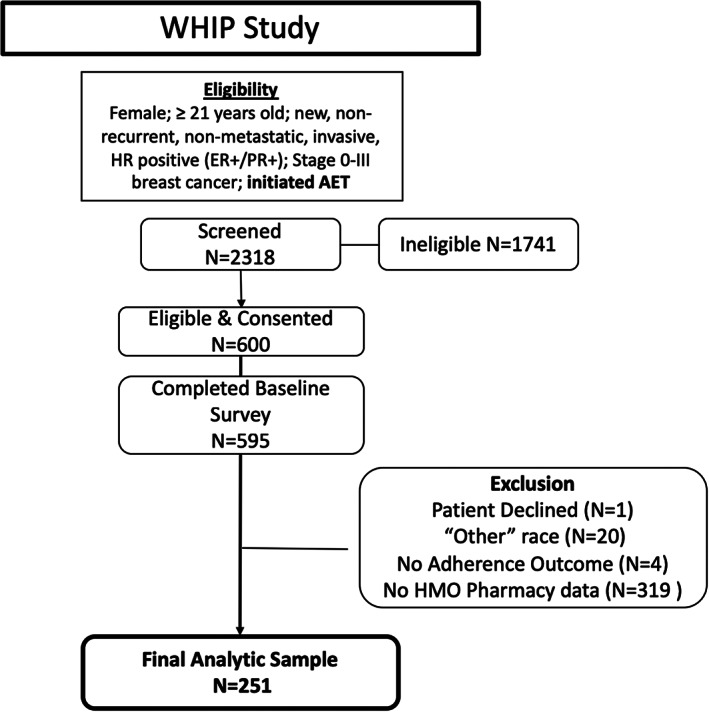
Recruitment and data collection process.

Among the 2318 women recruited in the WHIP study, 600 were eligible and consented through telephone surveys administered by trained clinical research assistants. Pharmacy fill and refill data for opioid prescriptions dispensed within 1‐year psotdiagnosis over the study period were reviewed and extracted for the final study sample. Women with complete pharmacy records for 1‐year postdiagnosis were included in the present study. The final sample of this secondary data analysis consists of 251 women. Screening, recruitment, and data extraction procedures adhered to the Strengthening the Reporting of Observational Studies in Epidemiology (STROBE) guidelines.^20^ The screening process is reported in Figure 1. The WHIP study was approved by the Institutional Review Boards at participating institutes. Study protocols adhered to the standards of the Health Insurance Portability and Accountability Act. Detailed methods and recruitment procedures pertaining to the present WHIP study can be found elsewhere.^18^, ^19^

### 2.2 Measures

At baseline, patients completed a self‐reported survey assessing demographics, cancer treatment history, evaluations on healthcare delivery, psychosocial measures, and symptom severity. We also obtained informed consent to extract saliva biospecimen data and medical and pharmacy records.

#### 2.2.1 Opioid prescription records

The types of opioids and duration of prescriptions were extracted from health records of breast cancer survivors (*n* = 251). We adapted the morphine milligram equivalent conversion calculator (MME) to classify the types of opioid medications supplied per patient.^21^, ^22^ The types of opioid medicines included mild, medium, or severe opioid medications. Based on the MME conversion values, severe opioids included oxycodone, methadone, or fentanyl; moderate opioids included morphine, hydromorphone, or hydrocodone; and mild opioids included use of codeine or tramadol. In our analysis, we classified opioid prescription status by the duration of opioid prescriptions supplied. A long‐term opioid prescription was defined as 90 days of consecutive opioid prescription supplied after diagnosis.

#### 2.2.2 Demographics and cancer treatment status

At baseline, we measured age, race/ethnicity, marital status, height/weight, income, employment status, and education. Information on cancer stage (defined by the American Joint Committee on Cancer [AJCC] staging system)^23^ as well as treatment types (chemotherapy [yes/no], radiation therapy [yes/no], hormone therapy [yes/no]) were extracted from medical records.

#### 2.2.3 Patient satisfaction on healthcare delivery

A prevalidated Patient Satisfaction Questionnaire Short‐form (PSQ‐18) was administered on a five‐point Likert scale to assess general satisfaction toward cancer care (e.g., “The medical care I have been receiving is just about perfect”), technical quality (e.g., “I think my doctor's office has everything needed to provide complete medical care”), interpersonal manner (e.g., “My doctors treat me in a very friendly and courteous manner”), communication (e.g., “Doctors are good about explaining the reasons for medical tests”), financial security/confidence (e.g., “I feel confident that I can get the medical care I need without being set back financially”), time spent (e.g., “Doctors usually spend plenty of time with me”), and access convenience (e.g., “I have easy access to the medical specialists I need”).^24^ Prior to computing composite scores for each subscale, several items were reverse‐coded to indicate that higher scores mean greater satisfaction toward cancer care and treatment, greater technical quality, better interpersonal manner, better communication, better financial security/confidence, less waiting time and more time with doctors, better time efficiency, and easier access.

#### 2.2.4 Beliefs about medicine questionnaire (BMQ)

The BMQ consists of two subscales: ‘perceived necessity of medication’ and ‘perceived concerns of taking the medication’.^25^ The perceived necessity subscale includes five items, such as “my current health depends on my hormonal therapy medications” and “without my hormonal therapy medications, I would become very ill.” Additionally, the perceived concern subscale includes five items on a five‐point *Likert* scale (e.g., “my hormonal therapy medications will protect me from becoming worse,” “I sometimes worry about the long‐term effects of my hormonal therapy medications”). Higher scores indicate greater perceived necessity and concerns.

#### 2.2.5 Medical outcomes study (MOS) social support survey

An eight‐item emotional support questionnaire (e.g., “Someone to confide in or talk to about yourself or your problems”) and a four‐item tangible support questionnaire (e.g., “someone to prepare your meals if you were unable to do it yourself”) were administered on a five‐point *Likert* scale. A higher score indicates having better emotional support and tangible support.^26^

#### 2.2.6 Functional assessment of cancer therapy‐breast [FACT‐B]

The prevalidated 37‐item FACT‐B instrument was implemented to measure multidimensional quality of life in patients with breast cancer, including the subscales for Physical Well‐Being (PWB), Emotional Well‐Being (EWB), Social/Family Well‐Being (SWB), and Functional Well‐Being (FWB), as well as 10 additional items of the Breast Cancer Subscale (BCS). The FACT‐B total score was computed in accordance with the FACT‐B scoring guidelines.^27^, ^28^, ^29^ Higher scores indicate greater overall well‐being and less health‐related concerns.

#### 2.2.7 Functional assessment of cancer therapy—endocrine symptoms (FACT‐ES)

A total of 23 items were administered to assess self‐reported concerns related to symptoms participants experienced in the past 7 days. Of those 23 items, 19 were adopted from the prevalidated FACT‐ES version 4 Additional Concern section. We also added three items to measure gastrointestinal symptoms (i.e., “I have experienced weight loss,”. “I have an increased appetite,” and “I have high cholesterol”) and one item to assess other concerns (i.e., “I have experienced bone loss”). Severity of endocrine symptoms and concerns regarding vasomotor (e.g., “I have night sweats,” “…hot flashes,” “…cold sweats”), neuropsychological (e.g., dizziness), gastrointestinal (e.g., “I have experienced weight loss”), gynecological and other symptoms (e.g., joint pain, stiffness) were measured on a five‐point *Likert* scale.^30^ Higher scores indicate worse symptoms (0 = not at all, 4 = very much).

#### 2.2.8 Psychological distress

At baseline, the level of distress was measured by the National Comprehensive Cancer Network's (NCCN) Distress Thermometer (DT) item on a 11‐point *Likert* scale. The DT item asked participants to “*indicate on a scale of 0 to 10, how you would best describe how much distress you have been experiencing in the past week including today. Zero represents no distress and ten represents extreme distress*.” Following the NCCN distress management guidelines, responses were categorized into three levels. Scores from 0 to 4 were coded as “1= Distress levels are well under control” scores from 5 to 6 were categorized to “2= Experience some distress”; and values 7 or higher were coded as “3= Experience high levels of distress.”^31^

#### 2.2.9 Social psychological factors

At baseline, patients were asked about religiosity with a nice‐item scale on a four‐point *Likert* scale (e.g., “I talk openly about my faith with others”)^32^; medical mistrust with seven items on a five‐point *Likert* scale (e.g., “Patients have sometimes been deceived or mislead by health care organizations”)^33^, ^34^; perceived discrimination in the medical system,^35^ using the prevalidated 14 items, where responses were on a binary scale (yes = 1, no = 0, e.g., “…been treated with less respect than other people because of your race”); and 12 items on a four‐point *Likert* scale assessing self‐efficacy in understanding healthcare and obtaining health information. Summated values were computed for religiosity, mistrust, perceived discrimination, and self‐efficacy. Higher scores indicate more religiosity, greater medical mistrust, greater discrimination perceived, and higher self‐efficacy.

### 2.3 Data analysis

To examine pain management outcomes stratified by race, we compared an array of factors and self‐reported outcomes between Black (*n* = 82) and White participants (*n* = 169). Next, to examine factors related to opioid prescription versus nonopioid prescription, patients who have not been prescribed opioids were categorized as ‘no opioid prescription’ (*n* = 79). The rest of the patients were categorized as patients with opioid prescription (*n* = 172). Lastly, we compared factors and outcomes related to long‐term opioid prescription. Ninety‐three patients fell under the long‐term (chronic) opioid prescription category (i.e., 90 days of consecutive opioid prescription after diagnosis). We conducted unequal‐variances t‐tests for continuous constructs and chi‐squared tests for categorical variables. We then performed a logistic regression model on opioid prescription status while controlling for covariates (age, income, BMI, marital status, cancer stage, and treatment received). Controlling for the covariates, we also conducted a logistic regression on chronic opioid prescription status. Surgery type was not added to the chronic opioid use model due to possible convergence failure that can be caused by the small number of cases within categories.^36^ As shown in Table 1, two of the categories reported 5 cases and 6 cases: each of these was less than 3% of the sample.

**TABLE 1:** Demographics and characteristics of study participants according to race (*N* = 251).

Overall	Total (*N* = 251)	Stratified by race		Test statistics
		Black (*n* = 82)	White (*n* = 169)	
Age, *M* (SD)	61.09 (11.07)	**58.84 (10.69)**	**62.18 (11.11)**	**−2.29**
Marital Status = Married (%)	149 (59.4)	**36 (43.9)**	**113 (66.9)**	11.13**
Income ≥100 k (%)	59 (25.2)	**8 (10.7)**	**51 (32.1)**	11.28**
Education ≤High school (%)	48 (19.3)	19 (23.2)	29 (17.4)	
Employment (%)				
Part‐time	36 (14.6)	12 (14.8)	24 (14.5)	
Full‐time	85 (34.4)	31 (38.3)	54 (32.5)	
Other	126 (51.0)	38 (46.9)	88 (53.0)	
BMI, *M* (SD)	29.75 (6.95)	**32.87 (7.37)**	**28.27 (6.24)**	4.54**
Cancer stage (%)				
I	151 (64.3)	**39 (51.3)**	**112 (70.4)**	8.33*
II	69 (29.4)	**31 (40.8)**	**38 (23.9)**	
III	15 (6.4)	**6 (7.9)**	**9 (5.7)**	
Surgery type (%)				
No surgery	5 (2.3)	3 (4.2)	2 (1.3)	
Lumpectomy	129 (58.1)	35 (49.3)	94 (62.3)	
Mastectomy	82 (36.9)	29 (40.8)	53 (35.1)	
Both	6 (2.7)	4 (5.6)	2 (1.3)	
Radiation = Radiation (%)	142 (74.0)	49 (81.7)	93 (70.5)	
Chemotherapy, *n* (%)	89 (35.6)	**40 (49.4)**	**49 (29.0)**	9.06**
Chronic Opioid Prescription, *n* (%)	93 (37.1)	**39 (47.6)**	**54 (32.0)**	**5.12**
Medication Tamoxifen, *n* (%)	75 (30.1)	29 (36.2)	46 (27.2)	
Adherence, *M* (SD)	0.94 (0.24)	0.90 (0.30)	0.95 (0.21)	
Activity, *M* (SD)	1403.65 (1771.62)	1101.42 (1853.34)	1547.66 (1719.02)	
Sitting, *M* (SD)	58.63 (30.58)	63.52 (36.13)	56.34 (27.44)	
Patient satisfaction questionnaire (PAQ‐18)				
General satisfaction, *M* (SD)	15.68 (3.10)	15.42 (3.21)	15.80 (3.06)	
Technical quality, *M* (SD)	12.10 (2.14)	**11.58 (2.17)**	**12.35 (2.09)**	−2.49*
Interpersonal manner, *M* (SD)	8.55 (1.28)	8.53 (1.21)	8.56 (1.31)	
Communication, *M* (SD)	8.12 (1.43)	8.06 (1.36)	8.15 (1.47)	
Financial aspect, *M* (SD)	7.45 (2.01)	**6.74 (2.23)**	**7.79 (1.80)**	−3.52**
Time spent, *M* (SD)	7.82 (1.41)	7.62 (1.49)	7.91 (1.36)	
Access convenience, *M* (SD)	12.43 (1.85)	12.25 (1.93)	12.52 (1.81)	
BMQ medication necessity, *M* (SD)	13.83 (3.08)	**14.41 (2.86)**	**13.55 (3.16)**	2.09*
BMQ medication concerns, *M* (SD)	11.28 (2.98)	11.69 (3.40)	11.07 (2.73)	
MOS emotional support, *M* (SD)	82.12 (19.81)	81.74 (19.35)	82.30 (20.08)	
MOS tangible support, *M* (SD)	80.23 (23.83)	79.83 (24.02)	80.43 (23.81)	
FACT‐B score, *M* (SD)	115.26 (17.32)	**109.14 (18.26)**	**118.18 (16.11)**	−3.59**
FACT‐endocrine subscale				
Vasomotor symptoms, *M* (SD)	4.05 (3.64)	**4.80 (3.60)**	**3.70 (3.62)**	2.13*
Neuropsychological symptoms, *M* (SD)	3.33 (3.20)	**4.20 (3.25)**	**2.93 (3.10)**	2.74**
Gastrointestinal symptoms, *M* (SD)	3.71 (3.49)	**4.53 (3.84)**	**3.33 (3.26)**	2.27*
Gynecologic symptoms, *M* (SD)	4.63 (3.92)	4.95 (3.65)	4.48 (4.04)	
Other symptoms, *M* (SD)	2.49 (1.93)	2.57 (1.77)	2.45 (2.03)	
Distress (%)				
Under control	153 (61.7)	47 (58.8)	106 (63.1)	
Some distress	61 (24.6)	19 (23.8)	42 (25.0)	
High distress	34 (13.7)	14 (17.5)	20 (11.9)	
Religiosity, *M* (SD)	28.80 (6.67)	**32.59 (4.14)**	**26.96 (6.90)**	8.03**
Mistrust, *M* (SD)	20.65 (4.70)	**23.02 (4.73)**	**19.51 (4.25)**	5.64**
Perceived discrimination, *M* (SD)	14.83 (2.41)	**16.12 (3.82)**	**14.20 (0.67)**	4.24**
Self‐efficacy, *M* (SD)	44.54 (3.66)	44.27 (3.57)	44.68 (3.71)	

*Note*: Values in bold *p* < 0.05. Values in bold and underlined *p* ≤ 0.01. Only significant statistical coefficients are reported.

Abbreviations: BMQ, beliefs about medicine questionnaire; MOS, medical outcomes study.

## 3 RESULTS

Among the screened (*n* = 2318), 600 patients were eligible and provided consent. Among 595 participants who completed the baseline survey, 344 were excluded due to incomplete health record data (see Figure 1 for detail), leading to the final sample of 251 patients. Most of our sample was White (*n* = 169, 67.3%) and married (*n* = 149, 59.4%), and the average age was 61.09 years old (*SD* = 11.07). Approximately 20% of the participants (*n* = 48) received a high school education or less, and 34.4% of the patients (*n* = 85) reported having full‐time employment (Table 1 for participant characteristics).

### 3.1 Opioid prescriptions

One hundred seventy‐two patients (68.5%) were prescribed opioids at least once, and 93 patients in the sample (37.1%) were on prescribed opioid medicines for 90 consecutive days or longer after diagnosis. Ninety‐seven patients (38.6%) were prescribed with severe opioids (i.e., oxycodone, methadone, fentanyl), and 63 were prescribed with these severe opioid medicines for 90 days or longer. More than one fourth of the patients in our sample were prescribed moderate opioids (i.e., morphine, hydromorphone, hydrocodone; *n* = 122, 48.6%), and 75 received long‐term moderate opioid medicines. Forty‐two patients (16.7%) were prescribed with mild opioids (i.e., codeine, tramadol), and 34 patients were on mild opioids for 90 days or longer. The average opioid prescription score was 2.50 (*SD =* 2.21). Black patients (*n* = 39, 47.6%) were more likely than White patients (*n* = 54, 32%) to be prescribed with opioid medicines for a long term (i.e., using 90 consecutive days after diagnosis), *Chi‐sq* = 5.12, *p* < 0.01 (Table 1).

### 3.2 Racial disparities in self‐reported outcomes

Black patients reported lower financial security and confidence in receiving medical care than Whites (*t* = −3.52, *p* < 0.1). Black patients (*M* = 118.18, *SD* = 16.11) were more likely than Whites (*M* = 109.14, *SD* = 18.26) to experience lower quality of life and more health‐related concerns as shown in FACT‐B scores (*t* = −3.59, *p* < 0.01). Additionally, Black patients were more likely than Whites to experience worse vasomotor (*t* = 2.13, *p* < 0.05), gastrointestinal (*t* = 2.27, *p* < 0.05) and neuropsychological symptoms (*t* = 2.74, *p* < 0.01). With regards to psychosocial outcomes, Black patients reported stronger religious belief (*t* = 8.03, *p* < 0.01), greater mistrust toward medical healthcare organizations (*t* = 5.64, *p* < 0.01), and greater perceived discrimination (*t* = 4.24, *p* < 0.01) than White patients (Table 1).

### 3.3 Differences in self‐reported outcomes by opioid prescription status

#### 3.3.1 Healthcare delivery

Patients who had mastectomy only or both mastectomy and lumpectomy were more likely to be prescribed opioid medicines for a long term than patients who received lumpectomy only (*Chi‐sq* = 16.90, *p = 0*.008). Nearly 50% of the patients prescribed with opioid medicines for 90 days or longer had received chemotherapy, whereas only 28.7% of the patients with no‐ or short‐term opioid medicines had chemotherapy (*Chi‐sq =* 8.07, *p* < 0.05, Table 2).

**TABLE 2:** Descriptive statistics stratified by opioid use status.

	Opioid prescription supplied			Chronic opioid prescription		
	No opioid prescribed	Opioid prescribed	t‐test	False	True	*t*‐test
	*n* = 79	*n* = 172		*n* = 158	*n* = 93	
Race = White (%)	**61 (77.2)**	**108 (62.8)**	**4.49**	**115 (72.8)**	**54 (58.1)**	**5.12**
Age, *M* (SD)	**64.09 (10.86)**	**59.72 (10.92)**	**2.96**	**63.01 (10.61)**	**57.83 (11.12)**	**3.63**
Marital status, married (%)	**39 (49.4)**	**110 (64.0)**		90 (57.0)	59 (63.4)	
Income, ≥100 k (%)	17 (23.3)	42 (26.1)		37 (25.7)	22 (24.4)	
Education, <high school (%)	14 (17.9)	34 (19.9)		32 (20.5)	16 (17.2)	
Employment (%)						
Part‐time	9 (11.4)	27 (16.1)		23 (14.7)	13 (14.3)	
Full‐time	23 (29.1)	62 (36.9)		48 (30.8)	37 (40.7)	
Other	47 (59.5)	79 (47.0)		85 (54.5)	41 (45.1)	
BMI, *M* (SD)	29.12 (7.29)	30.08 (6.77)		29.45 (7.41)	30.31 (6.04)	
Cancer stage (%)						
I	41 (55.4)	110 (68.3)		97 (65.1)	54 (62.8)	
II	27 (36.5)	42 (26.1)		43 (28.9)	26 (30.2)	
III	6 (8.1)	9 (5.6)		9 (6.0)	6 (7.0)	
Surgery type (%)						
No surgery	1 (1.6)	4 (2.5)		**2 (1.5)**	**3 (3.4)**	**16.90**
Lumpectomy	40 (63.5)	89 (56.0)		**93 (68.9)**	**36 (41.4)**	
Mastectomy	21 (33.3)	61 (38.4)		**38 (28.1)**	**44 (50.6)**	
Both	1 (1.6)	5 (3.1)		**2 (1.5)**	**4 (4.6)**	
Radiation = radiation (%)	41 (70.7)	101 (75.4)		93 (78.2)	49 (67.1)	
Chemotherapy = chemotherapy (%)	25 (31.6)	64 (37.4)		**45 (28.7)**	**44 (47.3)**	**8.07**
Medication tamoxifen, n(%)	20 (25.6)	55 (32.2)		46 (29.3)	29 (31.5)	
Adherence, *M* (SD)	**0.97 (0.16)**	**0.92 (0.27)**	**2.04**	0.96 (0.21)	0.90 (0.30)	
Activity, *M* (SD)	1479.11 (1880.43)	1364.62 (1718.06)		1345.01 (1609.41)	1512.54 (2046.00)	
Sitting, *M* (SD)	57.22 (30.38)	59.35 (30.76)		58.19 (29.99)	59.47 (31.85)	
PAQ‐18						
General satisfaction, *M* (SD)	15.89 (2.94)	15.56 (3.19)		**16.08 (2.80)**	**14.92 (3.50)**	**2.53**
Technical quality, *M* (SD)	12.17 (1.96)	12.06 (2.23)		12.32 (1.94)	11.69 (2.42)	
Interpersonal manner, *M* (SD)	8.53 (1.33)	8.56 (1.26)		8.61 (1.26)	8.45 (1.32)	
Communication, *M* (SD)	8.22 (1.38)	8.06 (1.46)		8.12 (1.41)	8.10 (1.48)	
Financial aspect, *M* (SD)	7.53 (1.80)	7.41 (2.11)		7.63 (1.90)	7.13 (2.17)	
Time spent, *M* (SD)	7.61 (1.52)	7.93 (1.34)		7.83 (1.44)	7.79 (1.36)	
Access convenience, *M* (SD)	12.42 (1.71)	12.44 (1.93)		12.52 (1.78)	12.27 (1.98)	
BMQ medication necessity, *M* (SD)	14.33 (3.15)	13.62 (3.04)		13.93 (3.04)	13.68 (3.16)	
BMQ medication concerns, *M* (SD)	11.31 (2.96)	11.26 (2.99)		11.03 (2.83)	11.69 (3.18)	
MOS emotional support, *M* (SD)	79.48 (20.27)	83.34 (19.53)		81.27 (21.39)	83.57 (16.75)	
MOS tangible support, *M* (SD)	76.28 (24.68)	82.07 (23.27)		78.19 (24.78)	83.72 (21.81)	
FACT‐B score, *M* (SD)	116.24 (17.30)	114.76 (17.37)		**117.20 (16.65)**	**111.65 (18.06)**	**2.25**
Endocrine subscale (ES)						
Vasomotor symptoms, *M* (SD)	4.33 (3.98)	3.90 (3.46)		3.97 (3.70)	4.19 (3.55)	
Neuropsychological symptoms, *M* (SD)	3.50 (3.37)	3.24 (3.11)		**2.97 (2.95)**	**4.01 (3.55)**	**−2.18**
Gastrointestinal symptoms, *M* (SD)	4.18 (3.89)	3.47 (3.26)		3.63 (3.49)	3.86 (3.51)	
Gynecologic symptoms, *M* (SD)	4.66 (4.62)	4.61 (3.52)		4.56 (4.12)	4.76 (3.55)	
Other symptoms, *M* (SD)	2.50 (2.02)	2.49 (1.89)		2.39 (1.96)	2.71 (1.88)	
Distress (%)			**6.08**			
Under control	**53 (67.1)**	**100 (59.2)**		101 (64.3)	52 (57.1)	
Some distress	**12 (15.2)**	**49 (29.0)**		34 (21.7)	27 (29.7)	
High distress	**14 (17.7)**	**20 (11.8)**		22 (14.0)	12 (13.2)	
Religiosity, *M* (SD)	28.33 (6.04)	29.02 (6.95)		28.75 (6.37)	28.88 (7.20)	
Mistrust, *M* (SD)	20.03 (4.47)	20.93 (4.79)		**20.00 (4.24)**	**21.73 (5.24)**	**−2.69**
Perceived discrimination, *M* (SD)	14.57 (2.28)	14.96 (2.48)		14.60 (2.10)	15.24 (2.87)	
Self‐efficacy, *M* (SD)	44.40 (3.79)	44.61 (3.62)		44.47 (3.75)	44.66 (3.53)	

Nonopioid users are those who have not been prescribed to opioid (no); opioid users are those who have been prescribed to opioid (yes); Values in bold with different superscripts indicate significant difference between two groups at a *p*‐value less than 0.05. Chronic opioid prescription is defined as 90 days of consecutive opioid supply after diagnosis.

*Note*: Values in bold *p* < 0.05. Values in bold and underlined *p* ≤ 0.01.

#### 3.3.2 AET symptom severity and psychosocial factors

We found that neuropsychological symptoms were significantly worse among patients prescribed with opioid medicines for 90 days or longer (*M* = 4.01, *SD* = 3.55) than those without opioid medicine prescribed (*M* = 2.97, *SD* = 2.95, *t* = −2.18, *p* < 0.05). Patients prescribed with opioid medicines for a long‐term reported significantly lower satisfaction toward the care (*t* = 2.53, *p* < 0.01), worse well‐being and more health‐related concerns (*t* = 2.25, *p* < 0.05), and greater mistrust (*t* = −2.69, *p* < 0.01) than those without prescribed opioid medicines (see Table 2 for details).

#### 3.3.3 Racial disparities in opioid prescription status and long‐term opioid prescriptions

Logistic regression models confirmed that racial disparity in opioid prescription status and long‐term opioid prescription remained. More specifically, the odds of opioid prescriptions in Black patients were 3.38 times that of opioid prescription occurring in White patients (CI: 1.46–8.36, *p* = 0.006) even after controlling for age, marital status, income, BMI, cancer stage, and chemotherapy status. The odds of chronic opioid prescription in Black patients were 2.72 times the odds of chronic opioid prescription happening in White patients (CI: 1.23–6.16, *p* = 0.014) after controlling for the same list of covariates (i.e., age, marital status, income, BMI, cancer stage, and chemotherapy status, Table 3).

**TABLE 3:** Logistic regression models.

Predictors	Odds ratios	95% CI	*p*
Opioid prescription			
Race [Black vs. White]	3.38	1.46–8.36	**0.006**
Age	0.96	0.92–0.99	**0.020**
Marital status [Married vs. Other]	2.87	1.42–5.97	**0.004**
Income [≥100 k vs. <100 k]	1.00	0.43–2.34	0.996
BMI	0.99	0.94–1.05	0.783
Stage [II vs. I]	0.37	0.16–0.82	**0.016**
Stage [III vs. I]	0.37	0.09–1.53	0.164
Chemotherapy [chemotherapy vs. no chemotherapy]	1.23	0.56–2.75	0.614
Adherence	0.44	0.06–1.88	0.320
Distress [some distress vs. no distress]	2.52	1.12–6.03	**0.030**
Distress [high distress vs. no distress]	1.02	0.40–2.71	0.969
Chronic opioid prescription			
Race [Black vs. White]	2.72	1.23–6.16	**0.014**
Age	0.95	0.91–0.98	**0.003**
Marital status [Married vs. Other]	1.66	0.81–3.51	0.175
Income [≥100 k vs. <100 k]	1.26	0.56–2.81	0.577
BMI	0.99	0.94–1.04	0.677
Stage [II vs. I]	0.50	0.22–1.12	0.101
Stage [III vs. I]	0.73	0.16–3.13	0.677
Chemotherapy [Chemotherapy vs. No Chemotherapy]	1.85	0.87–3.99	0.111
General satisfaction	0.91	0.81–1.02	0.104
FACT‐B score	0.99	0.96–1.02	0.381
Neuropsychological symptoms	1.02	0.90–1.16	0.713

*Note*: In these models, we controlled for age, marital status, income, BMI, cancer stage, and chemotherapy status. Values in bold are significant *p*‐values.

## 4 DISCUSSION

Opioid prescribing rates in cancer survivors are relatively high even after attaining 10 or more years of cancer survivorship.^5^ Opioids can be effective in treating acute and severe pain, but adverse effects among breast cancer survivors that commonly occur with opioid use are alarming.^37^ Moreover, our secondary data analysis indicates possible overprescribing of opioids among Black breast cancer patients. We analyzed health record and survey data of breast cancer patients who received adjuvant endocrine therapy (i.e., aromatase inhibitors vs. Tamoxifen) and identified demographic, healthcare delivery, symptomatic, and psychological characteristics associated with exposure to opioid treatments (e.g., chronic vs. nonchronic prescriptions) and race (Black vs. White). More than two‐third of patients had at least one prescription of opioids within the past 12 months, and 54% of them were prescribed opioid medicines for 90 consecutive days or longer after diagnosis. We found that overall breast cancer patients who were prescribed opioid medicines for a long term were more likely to report dissatisfaction, mistrust, and poor neuropsychological outcomes compared to those who were not exposed to opioid medicines. Some prior work reported associations between chronic opioid use and adverse effects, such as mental fogginess, central sleep apnea, and opioid‐induced hyperalgesia in noncancer patients.^38^, ^39^ Yet, empirical evidence for benefits and risks of long‐term opioid treatments in breast cancer patients is largely limited. To best of our knowledge, our findings specific to breast cancer patients reporting associations between chronic opioid prescription and neuropsychological outcomes as well as attitudes toward healthcare system are novel, thus contributing to the literature.

We examined racial disparities in pain management between Black and White breast cancer survivors. Our regression models suggest that the odds of short‐term opioid prescription as well as chronic opioid prescriptions were significantly higher among Black patients compared to White patients, even after controlling for covariates. In the current literature, findings on whether Black cancer patients tend to receive higher doses and more potent opioid prescriptions compared to other races are mixed. On the one hand, a study reported the opposite of what we found in our analysis.^40^ Using the Veterans Affairs (VA) Informatics and Computing Infrastructure (VINCI) nationwide database containing 106,732 military veteran cancer survivors diagnosed between 2000 and 2015, Vitzthum and colleagues found that white race was associated with the risk of increased adjusted odds of persistent opioid prescriptions received after the onset of curative treatment.^40^ In this dataset, other factors associated with persistent opioid prescriptions include younger age, unemployment at the time of cancer diagnosis, lower income, increased comorbidity, and current or prior tobacco use. On the other hand, Meghani et al.^41^ found that African American patients with cancer pain were more likely than White counterparts to receive morphine with toxic metabolites (i.e., morphine 3‐ and 6‐glucuronide), which are known to be neurotoxic and attribute to chronic kidney disease.^41^ Meghani et al. also reported that African American patients with cancer were more likely to be prescribed morphine‐based medications but less likely to be prescribed oxycodone‐based medications than White patients with cancer.

These mixed findings can be due to variation in characteristics of the selected samples, research settings where data were obtained, or variables that influence the direction and magnitude of the association between race and the level of opioid prescriptions. Conducting a meta‐analysis of findings related to racial disparities in opioid prescriptions received as well as understanding mechanisms of disproportionate opioid‐related treatments and adverse effects by race will help further elucidate disparities in cancer pain treatment and survivorship outcomes.

We also found that Black patients were more likely to report worse symptomatic and psychological outcomes compared to White counterparts: Black patients reported worse neuropsychological, vasomotor, and gastrointestinal symptoms, as well as lower FACT‐B scores (indicative of poor physical, social, emotional, and functional well‐being) and greater perceived discrimination and mistrust than White patients. In our study, the vasomotor symptom disparity mean ratio between Black and White participants was 29.7, and the neuropsychological symptom disparity mean ratio was 43.3. In the current literature, however, there is limited evidence reporting racial differences in vasomotor and neuropsychological symptoms among breast cancer survivors. One study reported that there was no difference in vasomotor symptoms between Hispanic and non‐Hispanic breast cancer survivors.^42^ According to O'Keefe and colleagues, in 2010 the breast cancer mortality disparity ratio between Black and White women with breast cancers was 41.8.^43^ Given this nationwide mortality disparity ratio, the disparity found in our study could be clinically meaningful.

Our findings suggest that racial disparities exist in symptomatic and psychological outcomes, requiring closer examination of the reasons for these disparities. The gap in vasomotor and neuropsychological symptoms between Black and White patients could be a contributing factor to the differences in gastrointestinal symptoms. Or, the worse neuropsychological, vasomotor, and gastrointestinal symptoms reported in Black patients could be due to environmental factors, such as limited access to high quality pain care and longer delay to treatment.^44^ Reasons for these disparities should be examined further in future studies.

Findings from our logistic regression models also raise concerns about the potential consequences of chronic opioid prescriptions that were more pronounced in Black cancer survivors than their White counterparts. Our findings imply unmet needs of pain management among Black cancer survivors who may have had limited resources for alternative treatments for pain management other than opioids. Given that exposure to chronic opioid prescriptions was associated with deficits in a wide range of symptomatic outcomes, cancer patients of racial minorities might be at an elevated risk to negative outcomes associated with chronic opioid therapy. These findings are consistent with the current literature on cancer pain management reporting that patients receiving persistent opioids medicines are at a greater risk for comorbidities and negative health outcomes.^41^, ^45^ Tan and colleagues found that breast cancer patients with pain who used opioids were significantly associated with nonadherence to vital adjuvant endocrine therapies and at greater risk for mortality after adjusting for adherence.^46^ Furthermore, as shown in our study, exposure to chronic opioid treatment was significantly associated with lower quality of life, psychological comorbidities, and greater concerns about health.

Other nonsignificant and mixed results noteworthy and counterinitiative include: the proportion of Black patients taking Tamoxifen (36.2%) was greater than that of White patients (27.2%) although statistically not significant (Table 1); and higher cancer stage was not always associated with chronic opioid exposure (Table 3). Given that tamoxifen is known to cause arthralgias,^47^ and patients with progressed cancer stage often need opioid medicines for cancer‐related pain, future research should further examine these associations.

Other than these pharmacologic approaches in treating cancer survivors, it will be beneficial to develop social and behavioral medicines for supportive care of cancer survivors, such as Petrocchi et al.'s smartphone‐based intervention to engage and empower breast cancer patients^48^ and Alavi et al.'s web‐based psychotherapy for mental health of cancer survivors.^49^ Social and behavioral medicines are concerned with patients' psychological, behavioral, and social needs and refer to integrating social and behavioral science knowledge and practices into treatment and prevention of illness to enhance the overall quality of patient care and life.^50^ Future research should focus on integrating principles of social and behavioral medicines to develop easily accessible interventions such as the aforementioned technology‐based behavioral therapies in treating pain and improving the overall quality of life of cancer survivors.

### 4.1 Limitations

Our study must be interpreted with limitations imposed by the study design and research setting. First, to be eligible participants had to be diagnosed with HR+ breast cancer within 1 year of survey enrollment. The extracted opioid prescription data included a history of opioids prescribed for up to 1‐year postdiagnosis. Within the up to 1‐year postdiagnosis period, we identified whether a participant was prescribed opioids (yes vs. no) and for how long (over 90 days or less). When a patient was prescribed opioids and how long after opioid prescriptions each patient took the survey varied by patients. Overall, the mean difference between first opioid prescription date and survey completion date was 288 days. However, it is possible some participants took the survey while actively taking opioid medications, whereas others were no longer taking opioid medicines when participating in the survey. Thus, self‐reported outcomes (e.g., symptoms) cannot be always interpreted as an immediate outcome of the exposure to opioids prescribed, and we did not make causality claims out of the findings, but associations. Future study should conduct a longitudinal follow‐up study with exact timestamp data between these activities to fully understand the consequences of chronic opioid exposure among breast cancer survivors.

Second, it should be noted that during the screening process, over half the sample was excluded due to incomplete HMO pharmacy records. Given our study objectives, individual‐level HMO data were necessary; thus, those without such data were considered ineligible. However, this exclusion could have invited potential biases in the results. To rule out any threat to sampling bias, future research should track if there is any demographic difference between the excluded sample and included sample.

Third, though one of the strengths of the study is the inclusion of data from multiple data sources (i.e., self‐reported patient surveys and health records), the sample size is relatively small for subgroup analysis after screening out ineligible patients. Future work may include a large enough sample size for diverse subgroup analyses by geosocial determinant factors, such as rurality versus urbanicity that previously demonstrated significant disparities in opioid overdose problems and treatment outcomes.^51^, ^52^, ^53^, ^54^ However, within our sample (*n* = 251), we still found significant differences across racial groups and several clinically important findings. Our analysis confirmed that breast cancer patients receiving chemotherapy were more likely to be on chronic opioid treatment than those who did not receive chemotherapy. Yet, the current dataset only reports a binary response of whether chemotherapy was given (yes or no). To further understand the granularity of chronic opioid use among cancer patients who underwent chemotherapy, it will be critical to assess the types and duration of chemotherapeutics given. Future research is required to address and validate this clinically important association by using a comprehensive dataset containing treatment variables such as the types of chemotherapeutics and AET as well as related biomedical markers.

Lastly, the study did not have a drug in‐take monitoring system that tracked opioid consumption, but rather used prescribing patterns extracted from pharmacy health records. With the present dataset, it is not known whether patients truly took the opioid medications they were prescribed. With this study condition, our statistical findings related to disparities should be interpreted as exposure to opioid medicines and opioid prescriptions supplied, rather than opioid medications consumed. Given that data on opioid in‐take was not available, future studies should implement a protocol that tracks opioid in‐take records.

## 5 CONCLUSION

There were racial disparities in pain management, symptoms, and quality of life associated with exposure to chronic opioid medicines. Among breast cancer patients with AET, those supplied with opioid medicines for 90 days or longer were more likely to report dissatisfaction, mistrust toward health system, and poor neuropsychological symptoms than those who did not receive long‐term opioid medicines. These differences between cancer survivors who were prescribed opioid medicines and those who were not emphasize the need for alternative nonpharmacologic approaches, such as supportive care by trained providers for managing post‐treatment pain and technology‐based interventions grounded in social and behavioral medicine sciences.

## AUTHOR CONTRIBUTIONS

**Sunny Jung Kim:** Conceptualization (lead); investigation (lead); methodology (lead); writing – original draft (lead); writing – review and editing (lead). **Reuben P. Retnam:** Formal analysis (lead); writing – review and editing (supporting). **Arnethea L. Sutton:** Data curation (supporting); investigation (supporting); project administration (supporting). **Megan C. Edmonds:** Data curation (supporting); project administration (supporting); writing – review and editing (supporting). **Dipankar Bandyopadhyay:** Formal analysis (supporting); supervision (supporting); writing – review and editing (supporting). **Vanessa B. Sheppard:** Conceptualization (supporting); funding acquisition (lead); supervision (supporting); writing – review and editing (supporting).

## Data Availability

Data available on request due to privacy/ethical restrictions: The data that support the findings of this study are available on request from the corresponding author. The data are not publicly available due to privacy or ethical restrictions.
